# Estimates of prevalence, time-trend, and association of smoking in adults living with HIV, HBV, and HCV (NHANES 1999–2018)

**DOI:** 10.1038/s41598-022-24291-6

**Published:** 2022-11-19

**Authors:** Jie Yang, Jin-Long Lin, Jing Liu, Xiao-Wen Jiang, Hao Zhang, Lei Peng

**Affiliations:** 1 Major Infectious Diseases Management Department, Public Health Clinical Center of Chengdu, Chengdu, 610066 China; 2 School of Marxism, Tsinghua University, Beijing, 100084 China; 3 Institute of Population Research, Peking University, Beijing, 100871 China; 4 People Liberation Army Haidian District 17th Retired Cadres Rest Home, Beijing, 100143 China; 5 Department of Epidemiology, School of Clinical Oncology, Peking University, Beijing, 100142 China; 6 Department of Social Medicine and Health Education, School of Public Health, Peking University, Beijing, 100191 China

**Keywords:** Diseases, Health care, Health occupations, Medical research, Risk factors

## Abstract

Although the smoking rate of human immunodeficiency virus (HIV), hepatitis B virus (HBV) or hepatitis C virus (HCV) infected people was much higher than that of the general population, smoking cessation interventions have long been ineffective. We aimed to examine the estimates of prevalence, time-trend, and association of smoking among people living with HIV, HBV, or HCV. This cohort was composed of 32,115 individuals from the NHANES database (1999–2018) and they were collected in the US. The time trend analysis of smoking and quitting rates was conducted using different years of survey follow-up and different infected groups. Multivariable logistic regression analysis was used to identify the risk factors related to smoking behavior of these infected people. Compared to non-infected smokers, infected smokers were more likely to be older (aged 30–39, OR = 9.92, CI 6.07–16.21; aged 40–49,OR = 3.51, CI 2.49–4.94), males (1.99, 1.54–2.55), lower education and economic level (1.78, 1.39–2.29; 2.05, 1.59–2.65), unemployed (1.63, 1.21–2.20), suffering depression (1.35, 1.05–1.72), and drug users (7.65, 5.04–11.59). Taken together, our study showed that these complex psychosocial characteristics and unhealthy behavioral factors might be major independent risk factors for increasing smoking rate and decreasing smoking cessation rate among these infected people.

## Introduction

The Global Burden of Disease (GBD, https://www.healthdata.org/gbd) project indicated that, among 20 + years old adults in the United States (US), the prevalence of Acquired Immune Deficiency Syndrome (AIDS) and deaths of liver cancer (caused by hepatitis virus) had been growing steadily from 1990 to 2019 (Supplementary Fig. S1). Although advanced medical methods could ensure the quality of life of infected people, such as antiretroviral therapy (ART) and gene therapy for human immunodeficiency virus (HIV)^1,2^, antiviral therapies for hepatitis B virus (HBV)^3^ and hepatitis C virus (HCV)^4,5^, the high smoking rate among infected people often led to poor efficacy^6–8^. For instance, tobacco smoking cessations among HIV and HCV infected people could increase the treatment efficiency by 76%^6^. In addition, compared to smokers without HIV, those with HIV had twice the risk of developing tuberculosis, a 6–15 year shorter lifespan^9^, and quitting smoking among people living with HIV decreased non-AIDS malignant-tumor by 34%, the risk of cardiovascular disease by 20%, as well as total mortality by 16%^10^. From the perspective of cellular biological mechanism, tobacco smoke could lead to oxidative stress and aggravate the pathogenesis of HIV by inducing cytochrome P450 (CYP) mediated metabolism and activating cigarette smoke components (like benzopyrene)^11^. Similarly, a case–control study also observed that higher smoking pack years were associated with higher hepatitis virus load, maintenance of high hepatitis virus load, more severe hepatotoxicity grades, and increased likelihood of alanine aminotransferase (ALT)^12^.

Although some studies had confirmed that smoking had a great negative impact on people living with HIV, HBV or HCV (HIV|HBV|HCV), there was still a lack of national large-scale sampling research to find the influencing factors, prevalence and time trend of smoking. Some studies reported the ratio of smoking rate among people infected with HIV. In Klerksdorp, South Africa, the smoking rate of males infected with HIV reached 52.3%^13^, 60.9% in Uganda^14^ and 55.4% in the US (Montefiore Medical Center, Bronx, New York)^15^. However, there was little study on the influencing factors of smoking and the time trend of smoking rate in people infected with HIV, HBV and HCV. We found only four small sample studies explored the risk factors behind smoking in patients with AIDS^16–20^. Because of the limited sample sizes in these single infectious disease studies, the calculated smoking rate might not be reliable. Thus, these results might be less representative and ungeneralizable to those with less healthcare access. Consequently, the intervention measures for tobacco control should be aimed at a larger group of different infectious patients in the US in order to be more effective and save labor force, material and financial resources.

A study showed that compared to general US people, prevalence of smokers living with HIV had been steadily higher from 1999 to 2016. Although the smoking rate in this population had decreased slightly over the past 20 years, smoking cessation interventions might have had little effect^21^. Hence, monitoring the smoking prevalence and changes in sociodemographic characteristics of the target population might be very important to reduce the smoking rate and disease burden of this population^21^. Similarly, another study showed that quitting smoking could improve the self-efficacy of individuals with substance use disorders^22^. However, few people succeed in quitting smoking, because smoking cessation outcomes might be related to depressive symptoms^23–26^, education level, poverty, unhealthy alcohol use, and drug use disorders^22^. The quantitatively description of correlates of smoking is now inconsistent and inconclusive.

In order to solve the problems not covered in the previous studies, this study used a nationally representative sample of American adults and relied on HIV antibody, HBV antigen, HCV RNA and blood test to determine the infection status (www.cdc.gov/nchs/nhanes/). The purpose of this current study was: (1) to compare demographic characteristics, psychological status, substance use, and smoking profile between people living with HIV|HBV|HCV and people without; (2) to analyze the change trend of smoking rate and smoking cessation rate of smokers living with HIV|HBV|HCV from 1999 to 2018; (3) to identify the relevant influencing factors of smoking among those living with HIV|HBV|HCV, mainly including sociodemographic characteristics, drug and alcohol abuse and mental health status. Analyzing and recording these data would be very necessary for assessing the severity of smokers living with HIV|HBV|HCV and guiding future smoking cessation interventions.

## Methods

### Data source

Data were obtained from the 1999–2018 National Health and Nutrition Examination Survey (NHANES). It was a nationally representative continuous queue survey database collected by the National Center for Health Statistics (NCHS), and employed a complex, multistage, probabilistic sampling design. The database was publicly available on the Internet and could be downloaded by researchers from all over the world. All details about the database could be efficiently acquired at www.cdc.gov/nchs/nhanes/. NHANES data have been collected every two consecutive years since 1999. All participants aged 21–59 who completed HIV antibody, Hepatitis B surface antigen, and Hepatitis C RNA blood assay testing and self-reported their current smoking status. Therefore, all participants aged 21–59 who had HIV antibody, Hepatitis B surface antigen, and Hepatitis C RNA blood assay test results (positive or negative) and self-reported cigarette smoking status (every day/someday) were included in the analysis. The final database for analysis consisted of three parts: demographics data, laboratory data, and questionnaire data.

### Measurements

#### HIV, HBV, and HCV diagnosis

HIV status was determined based on the HIV-1 antibody blood assay test results in NHANES. According to the test results, the subjects were divided into two groups: HIV + (people living with HIV) or HIV− (people without HIV infection).

The VITROS Hepatitis B surface antigen (HBsAg) test was performed by NHANES using the VITROS HBsAg Reagent Pack and VITROS Immunodiagnostic Products HBsAg Calibrator on the VITROS ECi/ECiQ Immunodiagnostic Systems and the VITROS 3600 Immunodiagnostic System. An immunometric immunoassay technique was used, which involves the simultaneous reaction of HBsAg in the sample with mouse monoclonal anti-HBs antibody coated onto the wells and a horseradish peroxidase (HRP)-labeled mouse monoclonal anti-HBs antibody in the conjugate. The bound HRP conjugate was measured by a luminescent reaction. The amount of HRP conjugate bound is indicative of the level of HBsAg present in the sample. Participants, based on the results, were placed into two groups: HBV + (people living with HBV) or HBV− (people without HBV infection).

NHANES used an in vitro nucleic acid amplification test (COBAS AMPLICOR HCV MONITOR Test, version 2.0) to quantitatively determine HCV RNA in human serum or plasma. According to the results of laboratory examination, subjects were divided into two categories: HCV + (people living with HCV) or HCV− (people without HCV infection).

#### Smoking

Smoking and quitting smoking states were assessed from two self-reported items in the data respectively: “Have you ever smoked cigarettes?” and “Do you now smoke cigarettes?”^21^ Smoking status was categorized into two groups: (a) persistent smokers who reported they smoked at least 100 cigarettes in their lifetime and currently smoke either every day or some days; (b) never smokers who reported they have not smoked 100 cigarettes in their lifetime, and don’t smoke now. Quitting smoking status was also placed into two parts: (c) former smokers, who have smoked at least 100 cigarettes in their life, being currently smoking every day or several days; (d) quitting smokers who attempted serious quitting during the past 12 months and stopped smoking for at least 1 day. The age at which smoking began came from this problem: “How old did you start smoking for the first time?” The smoking ratio was defined as the number of former smokers (a) to the number of persistent smokers (a) and never smokers (b). Similarly, the quit ratio was defined as the number of quitting smokers (d) to the number of former smokers (c).

#### Sociodemographic characteristics

Participants reported their age, gender, race (Hispanic, non-Hispanic White, non-Hispanic Black, other race), education level (high school or less, some college or more), marital status (married/living with partner, divorced/widowed/separated, never married), employment status (employed, unemployed), and health care insurance (insured, uninsured). Family poverty index ratio (PIR) was used to identify participants as living at either above (PIR ≥ 1) or below the economic level (PIR < 1). PIR was measured through the poverty guidelines from NHANES.

#### Depression and mental health

This study used the depression scale to evaluate the mental health status of participants, and depression was measured using the *Patient Health Questionnaire* (PHQ-9), which was used to determine the extent of depressive symptoms in the past 2 weeks. According to the sum score of nine items on the questionnaire, participants were divided into two groups: having no depression (score 1–4) or having mild/moderate/moderately severe/severe depression (score 5–27).

#### Substance use

History of substance use behaviors included: (1) heavy drinking history (defined as having 5 + (for males) or 4 + (for females) drink/day, every day, at any point in the past); (2) use of marijuana, hashish, cocaine, heroin, methamphetamine, and injection drugs.

### Statistical analyses

Data processing, statistical analysis, and graphic drawing were carried out with SAS version 9.4 (SAS Institute Inc., Cary, NC), R version 4.0.2 (http://www.R-project.org, The R Foundation), IBM SPSS version 22.0 and OriginPro version 2021. We stratified the pooled sample into different two groups (a. People living with HIV +, People living with HIV−; b. People living with HBV +, People living with HBV−; c. People living with HCV +, People living with HCV−; d. People living with HIV +|HBV +|HCV +, People living without HIV + &HBV + &HCV +) and examined group differences in sociodemographic characteristics, smoking profile, depression, mental health and substance use history. We combined 1999–2018 survey cycle data based on NHANES guidelines. Each person was counted as one observation each time in the merged dataset. Independent sample t-test was used for all continuous variables that obey normal distribution and the chi-square test for all categorical variables (Supplementary Table S1). We calculated the smoking rate and smoking cessation rate of all the above groups and plotted the trend line (Figs. 2, 3). Four multivariable logistic regression models were performed (Supplementary Table S2). These four models separately took ‘people living with HIV who are current smokers compared to people who are not smokers’, ‘people living with HBV who are current smokers compared to people who are not smokers’, ‘people living with HCV who are current smokers compared to people who are not smokers’ and ‘people living with HIV|HBV|HCV who are current smokers compared to people who are not smokers’ as outcome variables to explore the correlation degree and direction between each independent variable and these dependent variables. The four models contained all sociodemographic characteristics (gender, age, marital status, race/ethnicity, health insurance, education level, and employment status), substance use (ever heavy alcohol use, ever use any drug involving marijuana, hashish, cocaine, heroin, methamphetamine, and injection drugs), and depression. The result of the collinearity diagnosis showed that there was no collinearity (variance inflation factor, VIF < 10) among the independent variables studied. Missing data were processed by complete case analysis, and participants with missing information were excluded from the analysis. The results of four regression models in this study were expressed with odds ratio (OR) as well as 95% confidence interval (CI). Cohen’s criteria for gauging small, medium and large effect sizes of R^2^ are 0.2, 0.5 and 0.8, which were calculated from Cohen’s d values^27^. We used a Benjamini–Hochberg false discovery rate (FDR) to correct for multiple hypothesis testing, which was the estimated proportion of the false discoveries made over the total discoveries at a given significance level^28^. Statistical significance would be considered when P values or adjusted P values are below 0.05 (two-tailed).

### Ethical approval

Ethical review and approval were waived for this study, since all the data from National Health and Nutrition Examination Survey was publicly accessible.

### Informed consent

Informed consent from all subjects was obtained by National Health and Nutrition Examination Survey.

## Results

**Figure 1:**
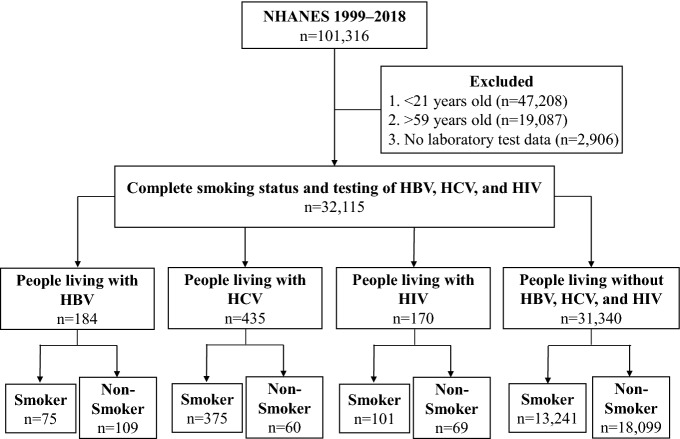
Study flow chart, NHANES 1999–2018.

There was a total of 101,316 people in NHANES database from 1999 to 2018. Among them, 47,208 participants younger than 21 years old, 19,087 older than 59 years old, and 2906 participants without laboratory test data were excluded. Overall, the remaining 32,115 participants with complete smoking status information and HIV, HBV, and HCV test results were included in the follow-up analysis (Fig. 1). Among people living with HIV, 101 were smokers and 69 were non-smokers; among people living with HBV, 75 were smokers and 109 were non-smokers; Among people living with HCV, 375 were smokers and 60 were non-smokers. Of those without HBV, HCV, and HIV, 13,241 were smokers and 18,099 were non-smokers.

### Characteristics of people living with HIV, HBV, or HCV compared to people without HIV, HBV, and HCV

Supplementary Table S1 showed the sociodemographic characteristics of the participants. Considering that the analysis between HIV, HBV or HCV infected persons and non-infected persons separately would lead to a decrease of statistical power, we combined the three groups of infected persons into one group to conduct difference test with the non-infected group. As a result, compared to smokers without HIV, HBV and HCV (HIV&HBV&HCV), people living with HIV|HBV|HCV were significantly older (40–49 years old, 34.5% vs. 26.1%; 50–59 years old, 38.5% vs. 22.7%), male (65.5% vs. 46.9%), non-Hispanic Black (37.0% vs. 20.5%), lower education level (High school or less, 57.7% vs. 45.7%), divorced/widowed/separated marital status (24.0% vs 14.4%), lower economic level (PIR < 1, 33.8% vs. 19.5%), unemployed (58.3% vs. 42.6%), uninsured (59.1% vs. 45.1%), heavy alcohol users (15.9% vs. 12.4%). In addition, HIV|HBV|HCV infected patients were more likely to suffer from depression (56.7% vs. 46.0%), using drugs including marijuana, hashish, heroin, methamphetamine, and injection drugs. Smoking prevalence was significantly higher among people living with HIV|HBV|HCV, with a younger age to start smoking regularly, compared to people without HIV&HBV&HCV (69.9% vs. 42.2%). The results shown above were statistically significant (all *p*-values < 0.05, two tailed).

### Time-trends in smoking prevalence and quit ratio

**Figure 2:**
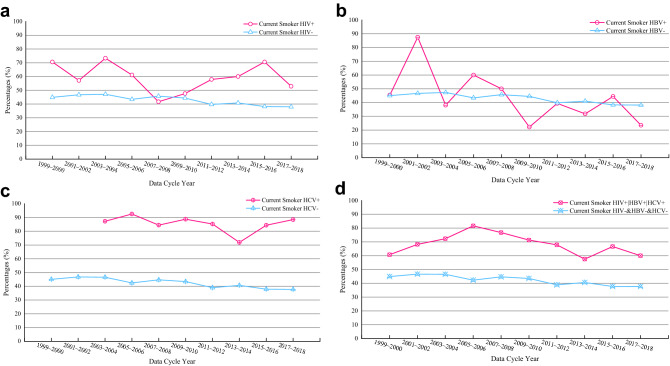
Trend analysis of change in the percentage of current smoking among people living with HIV|HBV|HCV compared to people without HIV|HBV|HCV (NHANES; 10 survey cycles, 1999–2018). (**a**) The percentage of current smoking between people living with HIV and without HIV; (**b**) the percentage of current smoking between people living with HBV and without HBV; (**c**) the percentage of current smoking between people living with HCV and without HCV; (**d**) the percentage of current smoking between people living with HIV|HBV|HCV and without HIV&HBV&HCV.

Figure 2 depicted the time-trend in smoking prevalence among people living with HIV|HBV|HCV compared to people without HIV&HBV&HCV from 1999 to 2018. Although the number of former smokers who were infected HIV +|HBV +|HCV + was much less than the number of those with HIV−| HBV−| HCV−, the time trend showed that the smoking rate of infected people was much higher than that of non-infected people over the 20-year period. For instance, among people living with HIV +, the current smoke rates ranging from 70.6% in 1999–2000 to 61.1% in 2005–2006 and from 57.9% in 2011–2012 to 52.9% in 2017–2018 were all higher than people with HIV− (Fig. 2a); among people living with HBV +, the current smoke rates (87.5% in 2001–2002 and 60% in 2005–2006) were higher than those with HBV− (Fig. 2b); among people living with HCV +, the current smoke rates changing from 2003–2004 (87.2%) to 2017–2018 (88.5%) were all higher than people with HCV− (Fig. 2c). Furthermore, we calculated the total smoking rate of HIV | HBV | HCV infected people, the time trend of smoking rate was the most stable (Fig. 2d), and the results showed that the smoking rate of infected people was significantly higher than that of non-infected people over the past 20 years.

**Figure 3:**
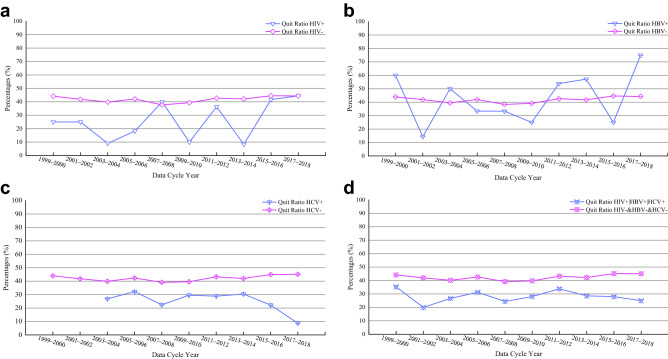
Trend analysis of change in the percentage of quit smoking among people living with HIV|HBV|HCV compared to people without HIV|HBV|HCV (NHANES; 10 survey cycles, 1999–2018). (**a**) The percentage of quit smoking between people living with HIV and without HIV; (**b**) the percentage of quit smoking between people living with HBV and without HBV; (**c**) the percentage of quit smoking between people living with HCV and without HCV; (**d**) the percentage of quit smoking between people living with HIV|HBV|HCV and without HIV&HBV&HCV.

Figure 3 depicted the time-trend in quit ratio among people living with HIV|HBV|HCV compared to people without HIV&HBV&HCV from 1999–2018. Similar to the results of smoking rate, the smoking cessation rate, over the 20-year period, also reflected the authenticity of Fig. 2 from the opposite side. In other word, the quitting smoking ratio of infected people was generally much lower than that of non-infected people. For example, among people living with HIV +, the quit ratios ranging from 25.0% in 1999–2000 to 18.2% in 2005–2006 and from 10.0% in 2009–2010 to 8.3% in 2013–2014 were all lower than people with HIV− (Fig. 3a); among people living with HBV +, the quit ratios (14.3% in 2001–2002, 25.0% in 2009–2010, and 25.0% in 2015–2016) were lower than those with HBV− (Fig. 3b); among people living with HCV +, the quit ratios ranging from 26.8% in 2003–2004 to 8.7% in 2017–2018 were all lower than people with HCV− (Fig. 3c). Moreover, we calculated the total quit ratio of HIV | HBV | HCV infected people, the time trend of quit ratio was the most stable (Fig. 3d), and the results showed that the quitting smoking ratio of infected people was significantly lower than that of non-infected people in the past 20 years.

### Influential factors of smoking in HIV|HBV|HCV infected people

In the first multivariable logistic regression model that compared to smokers without HIV, smokers living with HIV were more likely to be older (30–39 years old, OR = 7.80, CI 2.98–20.39), male (6.93, 3.33–14.44), non-Hispanic White (0.30, 0.14–0.68), and never married (0.44, 0.24–0.81). In the second multivariable logistic regression model that compared to smokers without HBV, smokers living with HBV were more likely to be non-Hispanic Black (0.09, 0.03–0.33) and drug users (3.29, 1.35–8.03). In the third multivariable logistic regression model that compared to smokers without HCV, smokers living with HCV were more likely to be older (30–39 years old, OR = 16.19, CI 7.96–32.91; OR = 3.74, CI 2.49–5.59), male (1.47, 1.11–1.96), lower education level (2.48, 1.83–3.36), lower economic level (1.96, 1.46–2.63), unemployed (1.86, 1.30–2.65), heavy alcohol users (1.67, 1.22–2.30), and drug users (14.15, 7.61–26.33). In the fourth multivariable logistic regression model that compared to smokers without HIV&HBV&HCV, smokers living with HIV|HBV|HCV were more likely to be older (30–39 years old, OR = 9.92, CI 6.07 to 16.21; 40–49 years old, OR = 3.51, CI 2.49–4.94), male (1.99, 1.54–2.55), lower education level (1.78, 1.39–2.29), lower economic level (2.05, 1.59–2.65), unemployed (1.63, 1.21–2.20), suffering depression (1.35, 1.05–1.72), and drug users (7.65, 5.04–11.59). Please see Supplementary Table S2 for the above data.

## Discussion

This study was the first research to combine HIV, HBV and HCV. The results showed that compared with the non-infected group, the smoking rates of the people infected HIV|HBV|HCV were 1.35–1.93 times higher, which was close to the results of other similar single infectious disease studies^29–33^. The smoking rates of infected people did not decrease significantly over the past 20 years, with an average of 68.3% being smokers. Among people living with HIV|HBV|HCV, correlates of smoking involved age, gender, race, education level, economic level, employment status, mental health status, and drug use history. These factors were not only the common characteristics of smoking in the target population, but also the deep root of their unhealthy behavior. It was worth mentioning that our study showed that depression and drug abuse were independent risk factors affecting the high smoking rate in this population, which was consistent with other relevant studies^29,34–37^. Currently, quite few intervention was able to address complex social and emotional needs that might affect this group's smoking behavior and response to smoking cessation interventions^38^. Therefore, strategies for the design of smoking cessation interventions should include the evaluation and treatment of depression and drug use, especially those infected with infectious diseases such as HIV, HBV, or HCV.

Especially among people living with HIV|HBV|HCV, smoking seemed to be a global public health issue that required special attention. The risky factors, such as older age, male, lower educational level, unemployed^39^, lower economic level^40^, depression^18,41^, and drug abuse^22^ were positively associated with smoking in our study, and the results were consistent with previous research reports from other countries around the world. Our results indicated that smoking behavior risk was significantly higher in HIV, HBV and HCV groups aged 30–49 years than in the younger age group. It was also understandable that the proportion of smokers in the male population infected with HIV, HBV and HCV was higher than that in women. The above results fully showed that the discrepancy of smoking rate in the subgroups of people infected with HIV, HBV and HCV was apparently affected by age and gender. In addition, the low socio-economic status might be an important risk factor that could not be ignored, because it could lead to the rise of smoking rate and the decline of smoking cessation rate and the low socio-economic status means that the education level was low or facing greater unemployment risk. Therefore, the unemployed, the people with low education level or low economic status might often received more false information about the harm of smoking. Also, Depression and drug use were common risk factors of smoking, and might be barriers to promoting health in groups facing health related challenges and having the least resources to deal with them. For example, some studies reported that smokers with AIDS would face health challenges, such as the high rate of substance abuse and high prevalence of mental illness in this group, such as depression, which would hinder the efforts of this group to quit smoking^42,43^. Interestingly, for the independent influencing factor of race/ethnicity, our results showed that the smoking rates of non-Hispanic White and Black infected HIV|HBV|HCV were lower than that of other races, which was contrary to a relevant study^21^. The possible reason was that whether Hispanic, non-Hispanic White or Black, some of their sociodemographic characteristics such as economic level (p > 0.05) were similar, or due to the low number of smokers among non-Hispanic blacks infected with HIV|HBV|HCV. Therefore, compared with infected individuals of other races, they had lower smoking rates.

Over the past 20 years, the time trend of smoking rates or smoking cessation ratios in both three single infectious disease groups and one integrated infectious disease group were consistent. This was also consistent with three previous research reports on HIV, which showed that the decline in smoking rate was not obvious in people living with HIV population^21,44,45^. The reason might be that the infected people had complex social psychological characteristics. For example, socio-economic challenges, mental illness (such as depression) and illicit drug use (such as marijuana and alcohol) might hinder their efforts to quit smoking. Therefore, there was an urgent need for targeted and tailored smoking cessation interventions to meet its unique profile and needs. Although the deep-seated reasons for the high smoking rate or low smoking cessation rate in the infected group needed to be further studied, our time trend study found a huge difference in smoking rate and smoking cessation rate between infected group and non-infected group, and intuitively showed their historical change law. In addition, we found that compared with the integrated multi-disease group, the smoking rate and smoking cessation rate in the single disease infection group were less easily distinguished from the non-infection group. Thus, this study showed that quitting smoking interventions for a variety of infectious patient groups rather than only for a single infectious disease group might be more effective and would greatly reduce the waste of public health resources.

When we analyzed a single infectious disease separately, it was not easy to obtain enough sample size and scientifically estimate the prevalence, time-trend, and association of smoking in adults living with HIV, HBV, or HCV. Therefore, the strength of this study was that skillfully combine HIV, HBV, and HCV infected people into an infected group to compare with the non-infected group, which might solve the problem of small sample size and obtained a robust time trend analysis result. However, there were some limitations to be further addressed. Prevalence study did not allow us to make any causal inferences about the observed associations, so we could only quantify the intensity and direction of correlation between smoking and its influencing factors. Though a strict quality control measures for the questionnaire data undertaken by the NCHS, the smoking survey information of participants in NHANES project was self-reported data, so it was inevitable to have reporting bias and measurement bias. Despite these limitations, the results of this study has provided a unique insight or enlightenment to solve serious public health problems on smoking of infected persons.

## Conclusions

HIV|HBV|HCV-infected smokers were at high risk for tobacco-related health problems and should become the target population of quitting intervention, for quitting smoking interventions for a variety of infectious patient groups rather than only for a single infectious disease group might be more effective. Although the smoking problem of this population was very serious, the current smoking cessation interventions did not significantly reduce the smoking rate. The time trend of smoking rate showed that the smoking rates of this population with HIV|HBV|HCV had been 1.35–1.93 times higher than the figures for non-infected people. This was because the unhealthy behavior of this smoking group might be affected by deep-seated factors such as some sociodemographic characteristics, depression, and drug use history. Compared to non-smokers with HIV|HBV|HCV, smokers were significantly older, male, lower education level, lower economic level, unemployed, drug users and suffering from depression. At present, our study has shown that complex psychosocial characteristics, individual economic status, mental diseases (such as depression) and illegal drug use (such as marijuana and heroin) might hinder their efforts to quit smoking. Hence, it was very essential to adopt targeted smoking cessation interventions to meet the unique situation and needs of this special group.

## Supplementary Information

Supplementary Information 1.

Supplementary Information 2.

## Data Availability

Data described in the manuscript, codebook, and analytic code will not be made available because the data used in this study were from the NHANES database, which is a free and open database for all researchers around the world. The link to the database is https://wwwn.cdc.gov/nchs/nhanes/Default.aspx.
