# Supplementary material for: Estimates of prevalence, time-trend, and association of smoking in adults living with HIV, HBV, and HCV (NHANES 1999–2018)
Source: Sci Rep. 2022 Nov 19;12:19925. doi: 10.1038/s41598-022-24291-6 (PMC9675830; doi:10.1038/s41598-022-24291-6)
Supplement: Supplementary file 1 — Supplementary Information 1. [file 41598_2022_24291_MOESM1_ESM.pdf]

Full Title: Estimates of Prevalence, Time-Trend, and Association of Smoking in Adults Living with HIV, HBV, and HCV (NHANES 1999–2018)

Authors: Jie Yang [1,#], Jin-Long Lin[2,3,#], Jing Liu[4,#], Xiao-Wen Jiang[5], Hao Zhang[6], Lei Peng[7,*]

1 Public Health Clinical Center of Chengdu, Chengdu 610066, China; yangjieHJ@gmail.com

2 School of Marxism, Tsinghua University, Beijing 100084, China; jllin@pku.edu.cn

3 Institute of Population Research, Peking University, Beijing 100871, China; jllin@pku.edu.cn

4 People Liberation Army Haidian District 17th Retired Cadres Rest Home, Beijing 100143, China; musihuangwu131@163.com

5 Department of Epidemiology, School of Clinical Oncology, Peking University, Beijing 100142, China; jxw1911110605@pku.edu.cn

6 Department of Social Medicine and Health Education, School of Public Health, Peking University, Beijing 100191, China; 2011210118@stu.pku.edu.cn

7 Department of Epidemiology, School of Clinical Oncology, Peking University, Beijing 100142, China

# These authors contributed equally to this work.

* Correspondence: 2011210593@stu.pku.edu.cn; Tel.: +86-138-0828-2849

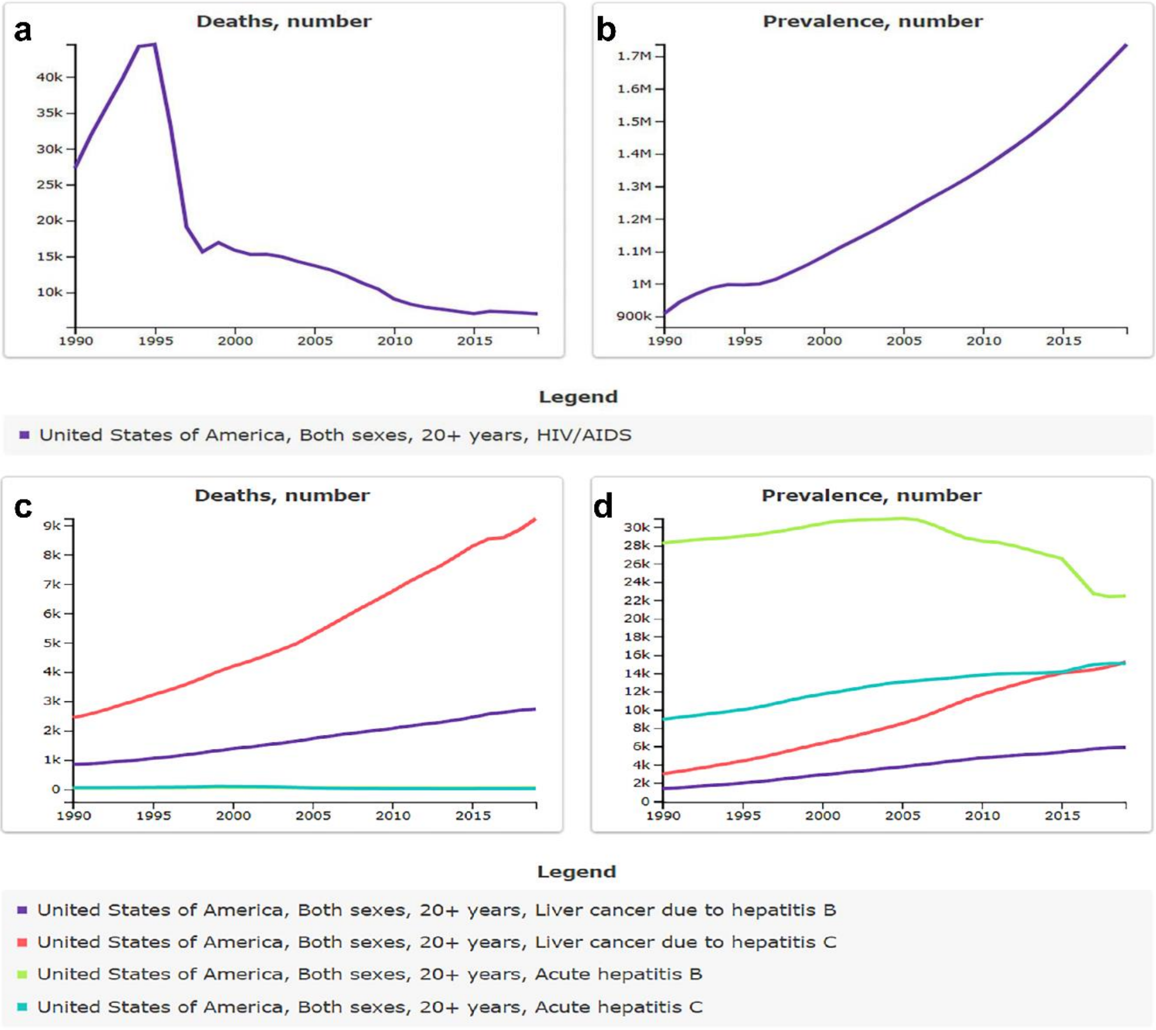

Supplementary figure S1. Analysis on the change trend of HIV, HBV, HCV, and liver cancer deaths and prevalence among US 20+ years adults from 1990 to 2019

a. the deaths of HIV/AIDS; b. the prevalence of HIV/AIDS; c. the deaths of liver cancer, HBV and HCV; d. the prevalence of liver cancer, HBV, and HCV.
