# Supplementary material for: Estimates of prevalence, time-trend, and association of smoking in adults living with HIV, HBV, and HCV (NHANES 1999–2018)
Source: Sci Rep. 2022 Nov 19;12:19925. doi: 10.1038/s41598-022-24291-6 (PMC9675830; doi:10.1038/s41598-022-24291-6)
Supplement: Supplementary file 2 — Supplementary Information 2. [file 41598_2022_24291_MOESM2_ESM.pdf]

Full Title: Estimates of Prevalence, Time-Trend, and Association of Smoking in Adults Living with HIV, HBV, and HCV (NHANES 1999–2018)

Authors: Jie Yang [1,#], Jin-Long Lin[2,3,#], Jing Liu[4,#], Xiao-Wen Jiang[5], Hao Zhang[6], Lei Peng[7,*]

1 Public Health Clinical Center of Chengdu, Chengdu 610066, China; yangjieHJ@gmail.com

2 School of Marxism, Tsinghua University, Beijing 100084, China; jllin@pku.edu.cn

3 Institute of Population Research, Peking University, Beijing 100871, China; jllin@pku.edu.cn

4 People Liberation Army Haidian District 17th Retired Cadres Rest Home, Beijing 100143, China; musihuangwu131@163.com

5 Department of Epidemiology, School of Clinical Oncology, Peking University, Beijing 100142, China; jxw1911110605@pku.edu.cn

6 Department of Social Medicine and Health Education, School of Public Health, Peking University, Beijing 100191, China; 2011210118@stu.pku.edu.cn

7 Department of Epidemiology, School of Clinical Oncology, Peking University, Beijing 100142, China

# These authors contributed equally to this work.

* Correspondence: 2011210593@stu.pku.edu.cn; Tel.: +86-138-0828-2849

Supplementary Table S1. Demographic characteristics, psychological status, substance use, and smoking profile among people living with HIV|HBV|HCV who are current smokers compared to people without HIV&HBV&HCV (NHANES 1999–2018)

	People with HIV (n=170)		People with HBV (n=184)		People with HCV (n=435)		People with HIV|HBV|HCV (n=775)		People without HIV&HBV&HCV (n=31,340)		Effect size	*P* value†
Characteristics	N	%	N	%	N	%	N	%	N	%		
Age (years)											0.084	<0.001
20–29	28	16.5	27	14.7	15	3.4	68	8.8	7,742	24.7		
30–39	47	27.6	43	23.4	54	12.4	142	18.2	8,319	26.5		
40–49	57	33.5	55	29.9	159	36.6	267	34.5	8,164	26.1		
50–59	38	22.4	59	32.1	207	47.6	298	38.5	7,115	22.7		
Gender											0.053	<0.001
Male	125	73.5	122	66.3	274	63.0	508	65.5	14,696	46.9		
Female	45	26.5	62	33.7	161	37.0	267	34.5	16,644	53.1		
Race/Ethnicity											0.046	<0.001
Hispanic	26	15.2	8	4.3	78	17.9	110	14.2	8,781	28.0		
Non-Hispanic White	29	17.1	15	8.2	210	48.3	250	32.3	12,876	41.1		
Non-Hispanic Black	112	65.9	57	31.0	126	29.0	287	37.0	6,440	20.5		
Other Race	3	1.8	104	56.5	21	4.8	128	16.5	3,243	10.3		
Education											0.045	<0.001
High school or less	83	48.8	87	47.3	285	65.5	447	57.7	14,332	45.7		
Some college or more	87	51.2	97	52.7	150	34.5	328	42.3	17,008	54.3		
Marital status											0.050	<0.001

Married/Living with partner	63	37.1	115	62.5	218	50.1	389	50.2	19,582	62.5		
Divorced/Widowed/Separated	27	15.9	31	16.8	130	29.9	186	24.0	4,522	14.4		
Never married	80	47.0	38	20.7	87	20.0	200	25.8	7,236	23.1		
Family poverty index ratio (PIR)											0.062	<0.001
<Poverty level (PIR<1)	56	32.9	39	21.2	173	39.8	262	33.8	6,115	19.5		
≥Poverty level (PIR≥1)	114	67.1	145	78.8	262	60.2	513	66.2	25,225	80.5		
Employment											0.055	<0.001
Yes	62	36.5	119	64.7	144	33.1	322	41.5	17,989	57.4		
No	108	63.5	65	35.3	291	66.9	453	58.3	13,351	42.6		
Health care insurance											0.048	<0.001
Yes	68	40.0	105	57.1	148	34.0	317	40.9	17,197	54.9		
No	102	60.0	79	42.9	287	66.0	458	59.1	14,143	45.1		
Have been heavy alcohol user [a]											0.027	0.003
Yes	17	10.0	10	5.4	98	22.5	123	15.9	3,874	12.4		
No	153	90.0	174	94.6	337	77.5	652	84.1	27,466	87.6		
Have depression [b]											0.066	<0.001
Score 1–4 Minimal depression	38	38.4	44	57.9	107	40.8	186	43.3	8,681	54.0		
Score 5–9 Mild depression	31	31.3	14	18.4	73	27.9	116	27.0	3,307	20.6		
Score 10–14 Moderate depression	11	11.1	5	6.6	36	13.7	51	11.9	1,280	8.0		
Score 15–19 Moderately severe	6	6.1	3	3.9	31	11.8	40	9.3	585	3.6		
Score 20–27 Severe depression	13	13.1	10	13.2	15	5.7	37	8.5	2,214	13.8		
Used marijuana or hashish											0.093	<0.001
Yes	77	68.8	46	40.4	305	86.6	420	73.9	10,727	53.0		

No	35	31.2	68	59.6	47	13.4	148	26.1	9,509	47.0		
Used cocaine											0.012	0.860
Yes	40	95.2	14	100.0	228	95.8	279	95.5	3,458	95.7		
No	2	4.8	0	0.0	10	4.2	12	4.1	157	4.3		
Used heroin											0.237	<0.001
Yes	7	16.7	2	14.3	117	49.0	126	43.2	395	10.9		
No	35	83.3	12	85.7	122	51.0	166	56.8	3,218	89.1		
Used methamphetamine											0.069	<0.001
Yes	18	42.9	6	42.9	129	53.8	153	52.2	1,374	38.0		
No	24	57.1	8	57.1	111	46.3	140	47.8	2,239	62.0		
Used a needle to inject illegal drug											0.326	<0.001
Yes	6	5.4	5	4.3	163	46.8	172	30.4	325	1.6		
No	106	94.6	110	95.7	185	53.2	393	69.6	19,939	98.4		
Smoking											0.149	<0.001
Yes	101	59.4	75	40.8	375	86.2	542	69.9	13,241	42.2		
No	69	40.6	109	59.2	60	13.8	233	30.1	18,099	57.8		
		**Mean±SD**		**Mean±SD**		**Mean±SD**		**Mean±SD**		**Mean±SD**		
Age of first using marijuana or hashish	77	18.1±5.7	46	18.8±5.3	305	15.4±4.0	420	16.2±4.6	10,718	17.4±4.5	0.004	0.676
Age of first using cocaine	40	23.8±6.5	14	25.0±7.2	228	22.3±6.6	279	22.7±6.6	3,455	21.7±5.7	0.001	<0.001
Age of first using heroin	7	30.0±10.7	2	26.0±4.2	115	23.5±8.0	124	23.9±8.2	394	24.0±7.7	0.001	0.514
Age of first using methamphetamine	18	26.2±10.8	6	33.2±10.6	129	23.8±8.5	153	24.4±9.0	1,373	22.2±6.7	0.008	<0.001
Age of first using cigarettes	101	17.8±4.9	75	19.2±5.5	375	16.6±6.0	542	17.2±5.8	13,241	17.8±4.7	0.001	<0.001

[†] Based on chi-square test or *t* test between people with HIV|HBV|HCV and those without; [a] 5^+ (males) or 4^+ (females) drink/day, every day, at any point in the

past; [b] Based on the Patients Health Questionnaire (PHQ-9); N, sample size; SD, standard deviation.

Supplementary Table S2. Multivariable Logistic Regression Analysis (NHANES 1999–2018)

	People living with HIV who are current smokers compared to people who are not smokers		People living with HBV who are current smokers compared to people who are not smokers		People living with HCV who are current smokers compared to people who are not smokers		People living with HIV|HBV|HCV who are current smokers compared to people who are not smokers	
	OR	95% CI	OR	95% CI	OR	95% CI	OR	95% CI
Age (years)								
20–29	1.00	—	1.00	—	1.00	—	1.00	—
30–39	7.80**	(2.98–20.39)	2.96	(0.95–9.27)	16.19**	(7.96–32.91)	9.92**	(6.07–16.21)
40–49	2.64	(1.27–5.50)	2.82	(0.99–7.93)	3.74**	(2.49–5.59)	3.51**	(2.49–4.94)
50–59	1.53	(0.76–3.01)	2.42	(0.83–7.08)	1.30	(0.95–1.76)	1.36	(1.04–1.79)
Gender								
Female	1.00	—	1.00	—	1.00	—	1.00	—
Male	6.93**	(3.33–14.44)	2.72	(1.17–6.32)	1.47*	(1.11–1.96)	1.99**	(1.54–2.55)
Race/Ethnicity								
Other Race	1.00	—	1.00	—	1.00	—	1.00	—
Hispanic	1.57	(0.63–3.93)	1.18	(0.29–4.87)	0.86	(0.59–1.25)	0.90	(0.64–1.26)
Non-Hispanic White	0.30*	(0.14–0.68)	0.80	(0.17–3.68)	0.84	(0.55–1.29)	0.64*	(0.45–0.93)
Non-Hispanic Black	1.80	(0.38–8.65)	0.09**	(0.03–0.33)	1.25	(0.62–2.49)	0.61*	(0.38–0.99)
Education								
Some college or more	1.00	—	1.00	—	1.00	—	1.00	—
High school or less	1.23	(0.70–2.16)	1.55	(0.64–3.75)	2.48**	(1.83–3.36)	1.78**	(1.39–2.29)
Marital status								
Married/Living with partner	1.00	—	1.00	—	1.00	—	1.00	—

Divorced/Widowed/Separated	1.60	(0.66–3.85)	1.21	(0.38–3.87)	0.73	(0.53–1.00)	0.81	(0.61–1.08)
Never married	0.44*	(0.24–0.81)	0.83	(0.32–2.15)	0.92	(0.63–1.35)	0.75	(0.55–1.02)
Family poverty index ratio (PIR)								
≥Poverty level (PIR≥1)	1.00	—	1.00	—	1.00	—	1.00	—
<Poverty level (PIR<1)	2.11	(1.17–3.79)	2.57	(1.03–6.42)	1.96**	(1.46–2.63)	2.05**	(1.59–2.65)
Employment								
Yes	1.00	—	1.00	—	1.00	—	1.00	—
No	2.34	(1.15–4.76)	0.41	(0.16–1.08)	1.86*	(1.30–2.65)	1.63*	(1.21–2.20)
Health care insurance								
No	1.00	—	1.00	—	1.00	—	1.00	—
Yes	1.04	(0.54–2.01)	0.89	(0.38–2.11)	1.39	(0.99–1.96)	1.26	(0.95–1.68)
Have been heavy alcohol user [a]								
No	1.00	—	1.00	—	1.00	—	1.00	—
Yes	1.45	(0.67–3.16)	2.56	(0.58–11.31)	1.67*	(1.22–2.30)	1.31	(0.98–1.74)
Having depression [b]								
Not at all	1.00	—	1.00	—	1.00	—	1.00	—
Yes (mild to severe depression)	2.24	(1.24–4.03)	1.43	(0.65–3.13)	1.13	(0.85–1.51)	1.35*	(1.05–1.72)
Any drug user [c]								
No	1.00	—	1.00	—	1.00	—	1.00	—
Yes	3.21	(1.48–6.95)	3.29*	(1.35–8.03)	14.15**	(7.61–26.33)	7.65**	(5.04–11.59)

**, adjusted $P<0.01$ *, adjusted $P<0.05$; [a] 5^+ (males) or 4^+ (females) drink/day, every day, at any point in the past; [b] Based on having a score 1–4: no depression, and a score of 5–27: mild to severe depression in Patients Health Questionnaire (PHQ-9); [c] Ever use of any drug use including marijuana, hashish, cocaine, heroin, methamphetamine, and injection drugs; OR, odds ratio; CI, confidence interval.
